# Whole-Genome Sequence of *Starmerella bacillaris* PAS13, a Nonconventional Enological Yeast with Antifungal Activity

**DOI:** 10.1128/genomeA.00788-17

**Published:** 2017-08-10

**Authors:** Wilson José Fernandes Lemos Junior, Laura Treu, Vinícius da Silva Duarte, Milena Carlot, Chiara Nadai, Stefano Campanaro, Alessio Giacomini, Viviana Corich

**Affiliations:** a Department of Agronomy, Food, Natural Resources, Animals and Environment, University of Padova, Legnaro, Italy; b Department of Environmental Engineering, Technical University of Denmark, Kongens Lyngby, Denmark; c Department of Microbiology, Universidade Federal de Viçosa, Viçosa, Brazil; d Department of Biology, University of Padova, Padua, Italy; e Interdepartmental Centre for Research in Viticulture and Enology, University of Padova, Conegliano, Italy

## Abstract

*Starmerella bacillaris* is a fermentative yeast commonly found in vineyards. Here, we present the draft genome sequence of *S. bacillaris* PAS13, a nonconventional enological yeast with a potential role as a biocontrol agent. This gene sequence will provide insights into the genetic basis of yeast activity against gray mold disease (*Botrytis cinerea*).

## GENOME ANNOUNCEMENT

To reduce the use of pesticides, biocontrol agents have been developed as potential alternatives to agrochemicals in integrated crop management (1). They can be a relevant part of an effective strategy to improve sustainable agricultural systems. In this context, several nonconventional yeasts, such as *Candida intermedia*, *Sporidiobolus pararoseus*, *Saccharomyces cerevisiae* (*Sa. cerevisiae*), and *Starmerella bacillaris* (*St. bacillaris*) (formerly *Candida zemplinina*) have been studied, given their ability to produce volatile organic compounds endowed with biocontrol activity against *Botrytis cinerea*. Moreover, *St. bacillaris* activity against gray mold has also been demonstrated *in vivo* on grape berries (2, 3). Furthermore, wine cofermentations (sequential or mixed inoculum) using *St. bacillaris* and *Sa. cerevisiae* have been widely investigated in recent years. These fermentations were characterized by the complementary consumption of glucose (by *Sa. cerevisiae*) and fructose (by *St. bacillaris*) with a consequent increase in glycerol and succinic acid contents, which is associated with low ethanol and acetoin production. This is an interesting feature for the improvement of wine quality and the reduction of ethanol content in wine (4–6).

*St. bacillaris* is commonly found in enological environments (7). Here, we present the draft genome sequence of *St. bacillaris* PAS13, isolated from destemmed dried grapes of the Raboso Piave variety, cultivated in the Bagnoli DOC (Guaranteed Origin Name) area in northeast Italy.

For genomic DNA extraction, zymolyase digestion followed by standard phenol-chloroform purification, as described by Vaughan-Martini and Martini (8), was used. Illumina 1-kb mate-paired libraries were prepared at the Ramaciotti Centre for Genomics (Sydney, Australia) and run on an Illumina NextSeq 500 platform. The sequencing resulted in a 147-fold genome coverage using 9,651,388 high-quality paired-end (2 × 150-bp) and unpaired reads. The *de novo* assembly was performed using SPAdes version 3.10 (with option -k 21,33,55,77,99,127) (9), generating a draft genome of 9.4 Mb with a GC content of 39.45%. The high quality of the assembly was proved by the presence of only 67 contigs in the genome (*N*_50_ length of 318,510 bp). GeneMark-ES software was used for predicting protein-coding sequences (CDSs) (10), and the results indicated the presence of 4,321 CDSs and 4,322 exons. According to Lemos Junior et al. (11), two different approaches were used for gene annotation, namely, BlastKOALA (12) and RPS BLAST. The first approach used members of the *Saccharomycetaceae* family as the taxonomy group to generate a nonredundant set of KEGG genes. The second approach was used to compare protein sequences with eukaryotic orthologous groups of proteins (KOG) (13). The *St. bacillaris* PAS13 genome reported here will help in understanding the metabolism of this yeast and its potential role as a biocontrol agent in vineyards.

### Accession number(s).

The whole-genome shotgun project of *St. bacillaris* PAS13 has been deposited in DDBJ/ENA/GenBank under the accession no. MWPI00000000. The version described in this paper is the first version, MWPI01000000.
